# Challenges in Metabolomics-Based Tests, Biomarkers Revealed by Metabolomic Analysis, and the Promise of the Application of Metabolomics in Precision Medicine

**DOI:** 10.3390/ijms23095213

**Published:** 2022-05-06

**Authors:** Alessandro Di Minno, Monica Gelzo, Marianna Caterino, Michele Costanzo, Margherita Ruoppolo, Giuseppe Castaldo

**Affiliations:** 1 Dipartimento di Farmacia, University of Naples Federico II, 80131 Naples, Italy; 2 CEINGE-Biotecnologie Avanzate, 80131 Naples, Italy; monica.gelzo@unina.it (M.G.); marianna.caterino@unina.it (M.C.); michele.costanzo@unina.it (M.C.); margherita.ruoppolo@unina.it (M.R.); giuseppe.castaldo@unina.it (G.C.); 3 Department of Molecular Medicine and Medical Biotechnology, School of Medicine, University of Naples Federico II, 80131 Naples, Italy

**Keywords:** metabolomics, biomarkers, professional and regulatory agencies, clinical practice, precision medicine, tailored treatments, cost of care

## Abstract

Metabolomics helps identify metabolites to characterize/refine perturbations of biological pathways in living organisms. Pre-analytical, analytical, and post-analytical limitations that have hampered a wide implementation of metabolomics have been addressed. Several potential biomarkers originating from current targeted metabolomics-based approaches have been discovered. Precision medicine argues for algorithms to classify individuals based on susceptibility to disease, and/or by response to specific treatments. It also argues for a prevention-based health system. Because of its ability to explore gene–environment interactions, metabolomics is expected to be critical to personalize diagnosis and treatment. Stringent guidelines have been applied from the very beginning to design studies to acquire the information currently employed in precision medicine and precision prevention approaches. Large, prospective, expensive and time-consuming studies are now mandatory to validate old, and discover new, metabolomics-based biomarkers with high chances of translation into precision medicine. Metabolites from studies on saliva, sweat, breath, semen, feces, amniotic, cerebrospinal, and broncho-alveolar fluid are predicted to be needed to refine information from plasma and serum metabolome. In addition, a multi-omics data analysis system is predicted to be needed for *omics*-based precision medicine approaches. *Omics*-based approaches for the progress of precision medicine and prevention are expected to raise ethical issues.

## 1. Introduction

The term “metabolomics” was first used at the beginning of this millennium to identify the area of functional genomics devoted to the analysis of metabolites [1,2]. Metabolomics defines the comprehensive characterization of small molecules derived from both the genome (i.e., endogenous metabolites) and their interaction with the environment (i.e., exogenous metabolites) [3]. In recent years, methods have advanced for metabolomics and have allowed the for reliable identification, detection, and quantification of new metabolites in food, plant, environmental, animal, and human research. The combined use of untargeted and targeted metabolomics has exhibited many advantages beyond analytical chemistry [4]. In addition to documenting the high hypothesis-generating potential [1], advancements in *omics* have provided significant information regarding new potential biomarkers [5]. Advanced data processing systems (e.g., informatics) have greatly helped to characterize metabolic pathways in different biological systems [6,7,8,9]. However, the possibility has also emerged that inherent technical limitations in analytical instrumentation and in methods of analyses might have slowed the progress and industrial applications of metabolomics [10,11]. How these shortcomings have been addressed is summarized in Section 2 of this review. Presently, easy and predictable quantification of metabolites is achieved in plasma or serum [6,12]. However, much work needs to be conducted to interpret and explore the overwhelming amount of data to date generated by metabolomics [13]. How to implement the relevance of metabolomics-based tests in biomedical research is discussed in Section 3 of this review. By integrating biomarkers with genetic and phenotypic characteristics that distinguish one patient from another with comparable clinical settings, precision medicine is aimed at systemically evaluating the underlying causes of disease so as to target health interventions to individual needs [14]. Translational opportunities of metabolomics are critical for the progress of precision medicine [13,14,15,16]. The extent to which the criteria applied to gather the information currently employed in precision medicine may help the advancement of metabolomics is discussed in Section 4 of this report.

## 2. Current Challenges in Targeted Metabolomics

Table 1 reports metabolomics-based biomarkers identified over the last decade in pre-natal and post-natal diagnosis, and in related experimental models by the authors of the present review. During the same time period, a variety of metabolomics-based biomarkers for characterizing environmental contaminants [17] or food derivatives [2], in addition to identifying the risk of diabetes mellitus [18,19], coronary heart disease [20,21,22,23] or cancer [24,25,26,27,28], have been identified. Advantages and disadvantages of different instrumental platforms, whose use is related to the chemical complexity of the biological system analyzed [10], have emerged in all these areas of metabolomics investigation. The fact [29,30] that very sensitive detectors (e.g., MS) that directly reveal very low concentrations of metabolites are not sensitive enough to simultaneously measure high-concentration components arose as a critical disadvantage. The need for different platforms and of different experts (analytical chemists, biologists, statisticians, data scientists and bioinformaticians) to achieve a comprehensive metabolome coverage [2,3] has also been recognized. Indeed, while reliably handling laboratory medicine issues, researchers trained in liquid chromatography–mass spectrometry (LC–MS) often need the help of experts in bioinformatics for the optimal experimental design for individual metabolomics studies and the appropriate statistics to be employed. This argues for large metabolomics groups with expertise and instrumentation sufficient to avoid contract laboratories (to carry out ad hoc experiments). A multifaceted research asset also enables to: (1) set up collaboration platforms with skilled metabolomics groups to increase chances to achieve funding for large program projects and overcome the high costs of analytical instrumentation, and (2) develop specialized training programs to teach beginners the broad spectrum of expertise needed for reliable analyses. How major additional pre-analytical, analytical, and post-analytical hurdles have been (and are being) addressed in metabolomics studies is summarized in the next few paragraphs.

- *Standard operating procedures*. The rationale for the wide spectrum of methods used in different metabolomics labs [11,31,32] stems from the following: (1) no single analytical method is sensitive and specific enough to allow for the identification and quantitation of the whole metabolome of even a single biological entity [31]; (2) the metabolome of a cell/organism contains metabolites differing in their concentrations (from g/L to pg/L) [31,33], turnover rates, and stability; and (3) the biology of different living organisms implies diversity in the metabolites to be identified/measured [1,33]. In view of this, the Metabolomics Society has set up the “metabolomics Standards Initiative (MSI)” Committee that has established rules to standardize metabolomics systems [2,34,35]. Quality control and standard operating procedures should be carefully followed to reduce pre-analytical errors [36]. Standardization of steps throughout the study procedure and data analysis (e.g., the analytical platform to be employed, instrumentation performance, the type of analysis to use, and the requirements for the interpretation of the output) prevent the risk of poor-quality control metabolomics protocols, incorrect quantification of metabolites, and deceptive data interpretation [35,37,38]. Few variables should be selected to make metabolomics data reliable [34].
- *Quantification.* Most of the data generated by metabolomics rely on normalization of the signal [3,30]. However, semi-quantitative approaches hamper multi-omics integration and translation of metabolomics data into clinical practice [11,39]. Definition of normal concentrations of a metabolite is key for early detection of pre-clinical conditions [40,41,42]. Consistent with the possibility that metabolomics can achieve absolute quantification of the metabolome [43], methods and analytical platforms for absolute quantification of the metabolome using targeted approaches are presently available [3,10]. Examples (Table 2) of technologies, platforms, and protocols for absolute quantification of several metabolites are now available [44,45,46,47,48,49,50,51]. Presently, the possibility is also documented that the relative, or absolute accuracy of quantification of newly discovered metabolites needs newer standardization steps [38,49,52].
- *Choice of separation methods.* Reverse phase approaches should be used for the separation of non-polar components (e.g., fatty acids), while normal phase approaches should be preferred to separate polar compounds (e.g., nucleotides and sugars) [11]. Thus, the compounds to be measured and the biochemical pathways to be identified define the separation method to be employed. Robust techniques (e.g., nuclear magnetic resonance, NMR) exhibit rather limited sensitivity of detections [15,53,54]. Advances in analytical instrumentation are overcoming such limitations [55,56]. The use of small-in-size NMR machineries and mass spectrometers provide wide coverage of metabolites [57]. Platforms with high reproducibility and detection consistency are being developed to reveal low concentrations of metabolites [57,58,59]. Two-dimensional chromatographic separations are becoming increasingly widespread [56], and MS based technologies are gradually being employed in a targeted fashion [51,60].
- *Combination of different techniques.* “Hyphenation” may be a new frontline in metabolomics [61]. Using standards or library spectra, spectroscopy produces selective information for identification of mixtures of chemical components separated by chromatography. Thus “hyphenation” combines the advantages of both techniques. Combinations of different techniques helps overcome limitations of single techniques and calls for major achievements in metabolomic studies [62]. Hyphenation of liquid chromatography–nuclear magnetic resonance–mass spectrometry liquid chromatography (LC–NMR–MS LC) has been developed for global metabolite profiling and identification of compounds [2]. The setup of such a platform needs to be simplified.
- *Statistical analysis.* A robust statistical analysis of the results (e.g., t-tests, ANOVA, principal component analysis [PCA], hierarchical cluster analysis (HCA), partial least square–discriminant analysis [PLSDA], volcano plots, correlation analysis) is critical for the reliability of metabolomics studies. For inherent reasons, the statistical significance for analytes that differ between cohorts is difficult to be determined in untargeted metabolomics. While enhancing the number of false negatives (type II errors), conservative approaches such as the Bonferroni correction limit false positive data (type I error) [4]. False discovery rate (FDR) approaches help address the issue of removing false positives, especially in untargeted metabolomics [63]. For instance, the Q-value calculates the maximally applicable correction to a given dataset [64].
- *Metabolite identification*. Both in targeted and untargeted metabolomics, the identification of “true” metabolites pushes upcoming steps of the analysis [65,66,67,68] and informative interpretation of features beyond the standard putative identification based on mass and/or retention time [69]. “True” metabolite identification is also critical for pathway analysis and mapping. The development of the human http://www.hmdb.ca/, accessed on 31 March 2022), food (http://foodb.ca/, accessed on 31 March 2022), DrugBank (https://www.drugbank.ca/, accessed on 31 March 2022) and T3DB metabolome database (http://www.t3db.ca/, accessed on 31 March 2022) helps achieve this goal. Especially for GC–MS methods [31,70] together with commercial metabolite libraries, in-house comprehensive spectral libraries of metabolites help convert putative metabolites/features into positive identifications [68]. Spectral libraries (e.g., Metlin or mzCloud) provide a reliable standard for the identification of the majority of naturally occurring metabolites present in biological materials [71], including those for which kits are not available [72]. Newer bioinformatics tools that employ web-accessed libraries are anticipated to improve automated metabolite identification [7,72].

## 3. Overpromising but Under-Delivering Translational Results

The high hypothesis-generating potential (and translational skills) of metabolomics is now established [41], and panels of biomarkers have been defined (http://www.mayomedicallaboratories.com, accessed on 31 March 2022). Using advanced analytical, community-based methods [72] and bioinformatics [1,5], (targeted) weaknesses in metabolomics have largely been overcome. Inherent technical limitations that might have delayed clinical and industrial translations of interfaces generated by this strategy have also been minimized. Accordingly, the whole human serum metabolome has been mapped in a UK population [73]. Additional issues are likely to be addressed through community-based approaches [16], and this may expand metabolomics-based opportunities to primary care facilities that have little access to expensive instruments [2], All this progress might be at odds [13] with the perception that metabolomics is overpromising but under-delivering translational results [15]. However: (1) other common biological matrices should be regularly explored, and (2) metabolomics information collected in clinical investigations should make a positive impact on the public [2]. In the present section, examples of how metabolomics-based research (as an emerging discipline) is currently being exploited to expand its role in health and disease are provided, and details on new potential directions to be pursued to improve our understanding of human pathophysiology are summarized.

- *Biomarkers discovery and validation*. Biomarkers are defined as objectively measured indicators of normal biological processes, pathogenic processes, or pharmacologic responses to a therapeutic intervention [5]. At variance with other biomarkers [13], metabolites are easily quantified at a low cost [41]. Most currently identified metabolomics-based biomarkers arise from studies that are rather limited in experimental designs [4], statistical robustness and validity [37]. Indeed, to date, biomarker discovery and validation has been often carried out in small uncontrolled trials [74]. Independent validation within the same topic, an attitude that increases confidence in the clinical strength of a potentially metabolomics-based test, is erratic. Because of the lack of a second evaluation in other cohorts, the possibility that any findings these studies have generated might be poorly reproducible should be considered. In keeping with this, a very limited number of the metabolomics-based biomarkers that have been reported to date, are widely employed in clinical practice [75]. Ad hoc, prospective trials are mandatory to validate biomarkers with high chances to impact clinical practice [76,77]. In this respect, numbers of patients to be tested may be limited in studies devoted to rare diseases [78], while they should be large enough in very common clinical settings (e.g., hypercholesterolemia). In the latter case, the possibility of identifying intermediate phenotypes (e.g., subjects with/without high lipoprotein levels in the circulation) should be considered.
- *Newer sources of metabolomic analysis*. The roadway of pathophysiology is key to understanding the machinery of diseases and to recognize the cause and the downstream effect of a disease. Investigations of metabolomics-based biomarkers should be carried out accordingly. Advancing towards this direction implies pathophysiology-oriented targeted metabolomics studies, which would be better if conducted in cooperation with other *omics* communities [79,80]. New sources of metabolomic analyses may be critical in this respect. In addition to plasma [81] and serum metabolome [82,83,84], discoveries in urinary metabolome [85,86,87,88] and in the volatilome, (e.g., breath) [89,90,91] will likely help gather/refine information on the mechanisms of major causes of death. Information-rich metabolomes may be also obtained from cerebrospinal fluid, human saliva, broncho alveolar lavage, sweat, feces, semen, and amniotic fluid. Studies struggle to extend measurements to intact tissues [4]. In clinical pharmacology, models of mammalian cultured primary cells are relevant for adsorption, distribution, metabolism, and excretion–toxicology (ADME–Tox) studies. With appropriate protections, the risk–benefit ratios of these studies may be defined for individual cases/diseases, and biomarkers identified [14,92].
- *Newer directions to be pursued.* Together with top causes of death in developed countries (ischemic heart and cerebrovascular disease, and malignancy) [4], the rapid rise of pathogens is acknowledged to increasingly contribute to world-wide mortality [93]. Newer pathogens are emerging [94]. ‘Traffic’ of microbes and the diseases they cause is facilitated in the globalized world of the third millennium. The adaptation into a new human host population may produce ‘new’ mutations in viruses, bacteria, or fungi that allow them to acquire new biological characteristics to adapt to new ecologies and to infect new hosts [95,96,97,98,99,100,101]. Pathogens may also be transmitted by human blood and blood-derived products. Donor selection and blood screening, and methods for their purification/inactivation have reduced the risk of pathogen contamination of blood/plasma-derived products and increased the safety of blood products [102]. However, the poor sensitivity/specificity of current screening methods, and the lack of reliable tests for some pathogens (e.g., prions), should be emphasized [94]. Metabolomics may minimize/eradicate the risk of contaminants in blood, including pathogens. Because of the rise in antimicrobial resistance, many normally harmless opportunistic microorganisms are increasing their pathogenicity, and bacterial infections are predicted to kill more humans than cancer and heart disease in the coming decades [103]. Metabolomics should work to establish ad hoc biomarkers to identify the appropriate strategy and prevent future deaths in the area of antimicrobial resistance.

## 4. Metabolomics in the Era of Precision Medicine

- *The promise of precision medicine.* Current clinical practice focuses on few variables and provides little information on their potential interactions. The identification of variables to classify individuals into sub-populations is critical for precision medicine and precision prevention [104]. Newborn screening for genetic mutations, e.g., phenylketonuria, and progress in dietary intervention to prevent the onset of diseases, are some of the earliest examples of precision medicine and precision prevention. Functional genomics has been the determining factor of an early tailoring approach once key profiles are identified [105]. More recently, information from genomics has been critical for the progress of cancer diagnostics, therapeutics, and prevention [106], and this way of thinking has been extended to the majority of areas in clinical medicine [14]. Cheap genome sequencing [107], powerful methods of functional genomics, large-scale biological databases, and computational tools for analyzing large sets of data have greatly fostered this attitude [108]. However, the genomic-approach based initiatives that have been launched in precision medicine to date, have delivered fewer disease genes than originally expected [3]. Limited information also arose from the approaches based on transcriptomics and everything relating to RNAs [109]. In keeping with this [110], evidence has emerged that: (1) many tumors are not genetically and metabolically homogeneous; (2) metabolic heterogeneity exists also within an individual tumor tissue [3], and (3) obesity-induced changes in adipose tissue microenvironment impact genetics of cardiovascular disease [111]. These limitations have shifted the attention from genomics-centered approaches to the impact of environment determinants in the initiation and progress of malignancy, and vascular disease [112]. At variance with genomics, which only foresees events based on genetic predisposition, metabolomics also reveals events related to gene–environment interactions [3]. As such, metabolomics is a key driver to exploring underlying pathogenic mechanisms of complex polygenic diseases (e.g., cancer, cardiovascular diseases, and diabetes mellitus) for which environmental factors (e.g., diet) substantially impact disease onset and development [113]. Recent improvements in single cell metabolomic analysis [114] lend credence to the possibility of specific treatments for individual metabolic microenvironments within diseased tissues [115].
- *How metabolomic information can potentially benefit the development of precision medicine: an example.* Cystathionine β-synthase deficiency (CBSD, EC 4.2.1.22), also known as homocystinuria (OMIM 236200, mean prevalence worldwide 1:335,000, ranges 1:1800–1:900,000), is a recessively inherited disorder of the catabolic pathway (the transsulfuration pathway) for the essential amino acid methionine (Met) [116]. Met is converted to the non-structural amino acid homocysteine (Hcy), via S-adenosylmethionine (SAM) and S-adenosylhomocysteine (SAH), by the release of a methyl group that is used in methylation reactions (e.g., via phosphatidylethanolamine N-methyltransferase, PEMT). CBSD impairs the conversion of homocysteine (Hcy) to cystathionine, leading to Hcy accumulation in plasma (up to 200 µm/L) and urine (homocystinuria) [117,118,119,120]. Severely affected patients with CBSD present ectopia lentis, learning difficulties, connective tissue disturbances including skeletal abnormalities (marfanoid habitus), osteoporosis, propensity to venous and arterial thrombosis, premature atherosclerosis and occasional liver steatosis. Presently, genotype–phenotype correlation in homocystinuria remains obscure [120]. Using an ultra-high-performance liquid chromatography–electrospray ionization–quadrupole time-of-flight–mass spectrometry method, and employing an untargeted lipidomic approach, we have identified a novel biochemical abnormality in plasma from 11 severe CBSD patients (belonging to nine unrelated families and carrying different genetic defects already reported in patients with CBS), consisting of a depletion of phosphatidylcholine (PC; *p* = 0.02) and lysophosphatidylcholine (LPC; *p* = 0.003) species containing docosahexaenoic acid (DHA), and a higher than normal medium and long-chain polyunsaturated fatty acids content in phosphatidylethanolamine (PE) and lysophosphatidylethanolamine (LPE) species (*p* < 0.02). This suggests impaired in vivo PEMT activity. As PEMT needs methyl groups to convert PE into PC, SAM and SAH were measured by LC–MS. Whole blood SAM and SAH concentrations were 1.4-fold (*p* = 0.015) and 5.3-fold (*p* = 0.003) higher in CBSD patients than in controls. A positive correlation between SAM/SAH and PC/PE ratios (*r* = 0.520; *p* = 0.019) was found. CBSD patients with liver steatosis (5/11) had a significantly lower PC/PE ratio than those without (48.26 ± 18.7 vs. 86.28 ± 14.4, respectively; *p* = 0.016). After correcting for age and gender, liver steatosis was associated with PE/PC ratio in a multivariate linear regression analysis (β = −0.770; *p* = 0.009) [78]. Pathophysiological information is that a diminished PEMT expression/activity as reflected by a decrease in hepatic PC/PE ratio, is consistently correlated with hepatic steatosis in mice [121]. SAH accumulation inhibits PEMT, and SAH-mediated impairment of PEMT is linked to hepatic steatosis [121,122]. Additionally, in a transgenic model (HO mice) that expresses very low levels of CBS and high plasma concentrations of Hcy and SAH, a post-translational repression of PEMT that inversely correlates with liver steatosis is present, together with upregulation and down-regulation of phospholipid species and SAM/SAH ratios similar to those found in our CBS patients [123,124]. Together, these findings in CBSD patients highlight the impact of Hcy levels on SAM/SAH levels regardless of the underlying genetic defect, arguing for directions to be pursued to understand the phenotypic heterogeneity of severely affected patients with CBS deficiency, and to provide guidelines to design innovative strategies in this area.
- *Metabolomics towards precision medicine*. Tough guidelines have been applied to design studies to attain (and analyze) data to be used in precision medicine and precision prevention approaches. The use of big biobanks and electronic medical records that integrate biological information with clinical data has strengthened and refined information from these studies [125]. Large, prospective, time-consuming and expensive studies are mandatory to validate older, and discover newer, metabolomics-based biomarkers with high translational chances [3]. This is predicted to uncover new pathological pathways and disease biomarkers, to improve disease prognoses, and facilitate treatment selection. To this end, information on metabolomics-based health data collection in families, and new imaging techniques to monitor changes in metabolite levels, are critical for the translation of metabolomics-based results into clinical and industrial application.
  ○ *Metabolomics-based health data collection in families.* In the second half of the last century, health data collection in “healthy” individuals and their families helped predict disease through the identification of biomarkers suggestive of pre-clinical conditions, and allowed for informative decision making and ad hoc preventive strategies [126]. Such measures had substantial economical and welfare effects (when supported by human validation studies), and provided large-scale biological databases to help predict post-treatment outcomes [108]. Metabolomics-based health data collection is likely to be critical for improved big data analysis and tailored medical decisions. For instance, citrate, an important biomarker of cancer [127], is increased in older healthy individuals [73]. A comprehensive information on citrate levels in healthy individuals of different ages is key for the progress of precision medicine (e.g., preventive screenings and early phases of a malignancy) [128].
  ○ *New imaging techniques to monitor changes in metabolite levels.* In the analysis of the data on CBSD patients summarized above, multi-dimensional scaling (MDS) analysis, based on lipid abundance, was implemented by the ‘DaMiRseq’ R/Bioconductor package [129] to identify specific clusters or batch effects. Differential analyses (CBSD patients vs. controls) were performed by the ‘limma’ R/Bioconductor package [130], implementing linear models adjusted for the effect of ‘Smoking’ [131,132]. The Benjamini–Hochberg procedure was used to control for the FDR. A lipid was deemed significant if the FDR adjusted *p*-value was <0.05 and the |log_2_(Fold Change)| > 1.5. Clustering analysis, performed by MDS showed that, except for smokers, CBSD and control groups were well separated both in positive and negative ion modes. In view of the key role of smoking in the top causes of death, the present example strongly supports the need for newer imaging techniques to strengthen the role of metabolomics in advanced research and avoid false overlapping in lipidomic analysis.
  ○ A high likelihood of *translation into a routine clinical test* argues for large cohort multi-center studies to validate metabolomics-based biomarkers with high chances to impact clinical practice. Healthy individuals should be seriously considered in new metabolomics studies. Indeed, metabolomics is predicted to be critical for developing medical devices that are unique to a patient (or small groups of patients). However, metabolomics-based data should also help develop devices for the health population (or for field testing of a disease) [4]. To this end, professional and regulatory agencies should provide updated robust guidelines for study design, data acquisition and validation, to be applied from the very beginning of a project [79,80,133]. Conversion of results into products is maximal when ad hoc plans and paths are defined at the start of a project. Upon completion of data acquisition, identification of mechanisms leading to a metabolic pattern increase the chances of successful translation of results into clinical and industrial application [11]. Perhaps together with suppliers involved in developing analytical platforms, the search for cheap and easy miniaturized instruments will be critical for smart modifications of biomarkers. In this respect: (1) methods for absolute quantification of a wide range of metabolites using easy analytical instrumentation should be implemented. Rather than targeted, special attention should be devoted to newer untargeted quantitative metabolomics methods; (2) new better platforms are needed to work together with other *omics* to progressively increase the number of genes that expose to (or protect from) illnesses; (3) efforts to evaluate the influence of confounding factors (e.g., age, gender, ethnicity, diet) on metabolomics results should be implemented [73], and (4) validation studies in health and disease are urgently needed to remove potential bias. Validation is especially mandatory in view of the: (a) inter-laboratory variation in techniques of different metabolomics institutions [4]; (b) lack of common practices to validate potential biomarkers, (i.e., the absence of generally accepted procedures for metabolic profiling for biomarker discovery); and (c) use of metabolomics data as a source of potential pharmacologically active compounds [134,135].

## 5. Conclusions

Over the centuries, changes in medical attitudes have dramatically improved the cure, and sometimes prevented the development of diseases (e.g., tuberculosis). The attitude of metabolomics to reliably analyze metabolites, and identify new biological matrices is now established, and technological and computational improvements have greatly enhanced the translational capability of this *omics*. The existing applications of metabolomics in precision medicine translate to advancements in the diagnosis, prevention, and treatment of disease. Improving instrumentation and implementing standard analytical procedures is predicted to strengthen the impact of metabolomics in future medical care. The roadway of precision medicine and precision prevention is likely to be critical to validate old, and identify new, metabolomics-based biomarkers. Information from sources other than plasma and serum, and advanced pathophysiological analysis will likely refine the picture of a disease based on measurements of the plasma or serum metabolome [136,137]. 

Considering the information gathered in genomics- and transcriptomics-based initiatives, it is predicted that precise clinical decisions and precision treatments will largely abide by the accuracy of the information available, that is largely *omics* in nature. Truly integrated multi-omics analyses have not been widely applied. Major effort is now mandatory to develop the analytical infrastructure required to generate, analyze, and annotate multi-omics data and inform decision-making in precision medicine. Broad incorporation of machine learning techniques and systems to provide doctors with fully automated clinical analyzers are likely to be needed to assist in disease diagnosis and treatment and predict prognosis in precision medicine [110]. Major hurdles that *omics* (first, metabolomics) will face in this new dimension are largely ethical. Firstly, predictive diagnosis will change the relationships among patients and healthcare providers, and increase physician visits, laboratory tests, and patient anxiety. Presently, the poor pathophysiological information about the overwhelming amount of data generated to date hampers translation of metabolomics to clinical practice. A systematic approach to determining (genetic) causality is mandatory. Secondly, using genomic, clinical, personal, and environmental data collected from very large numbers of individuals from various populations, and connecting their health records, “non-responders“ to a treatment, might belong to definite minority populations An effort is needed against discrimination in access to treatments [125]. 

**Table 1:** Examples of information collected employing targeted and/or untargeted metabolomics approaches in experimental models of disease and in pre- and post-natal diagnoses in humans.

Models of Disease		
Source of Material	Main Findings	Refs.
*Human, adult*	Dysregulation of lipid metabolism and pathological inflammation in patients with COVID-19.	[138]
	Liver abnormalities involving carbon and nitrogen metabolism in moderate and severe COVID-19 patients	[139]
	Plasma phospholipid dysregulation in patients with cystathionine-beta synthase deficiency ^§^	[78]
	Plasma levels of platelet-activating factor and its precursors in patients with familial hypercholesterolemia on treatment with PCSK9 inhibitors ^§^	[22]
	In vivo thromboxane A_2_ biosynthesis and endothelial function in patients with familial hypercholesterolemia receiving PCSK-9 inhibitors therapy ^§^	[140]
*Human, pediatric*	Serum phospholipid profile allows for the discrimination of infants who develop celiac disease before 8 years of age	[141]
*Animal*	A targeted metabolomic approach to a mouse model of mucopolysaccharidosis IIIB identifies specific amino acid and fatty acid metabolic pathway alterations	[142]
	Mice model of Glutaric aciduria type I (GA-I, OMIM # 231670), an inborn error of metabolism caused by a deficiency of glutaryl-CoA dehydrogenase. *	[143]
**Reference Values as Related to Gender Differences**		
*Human, adult*	Serum metabolomic profiles suggest influence of sex and oral contraceptive use.	[144]
*Human, pediatric*	Effect of gender on human premature blood metabolome in neonates.	[145]
	Effect of gender on urinary excretion of organic acids in children. °	[146]
	Effect of gender on blood metabolome of female and male human babies.	[147]
*Animal*	Effect of gender on amino acid and carnitine levels in rat tissues (heart, liver, kidney)	[148]

* Gaining insights into (brain) pathophysiology, and the development of new therapeutic interventions. ° relevance of analyzing human metabolome. ^§^ untargeted metabolomics, combined metabolomic and lipidomic approach.

**Table 2:** Examples of absolute quantification of metabolites using targeted approaches: source of metabolites, available methods and analytical platforms employed.

Type, (Numbers), and Source of Metabolites Quantified	Quantification Method	Platform	Refs.
Amino and non-amino organic acids (67), urine and serum samples.	MCF derivatization	GC-MS/MS	[46]
Polar primary metabolites (49), chickpea cultivars	BSTFA derivatization of primary metabolites	GC-MS	[149]
Amino and non-amino organic acids (50–100, human biological samples).	Calibration curve-free GC–MS method using MCF	GC-MS	[150]
Amino metabolites (124), renal cancer tissue, rat urine and plasma.	Derivatization assisted sensitivity enhancement with *5*-AIQC	UPLC-MS/MS	[151]
Lipids, lipidomic quantification (222), human serum samples.	PRM	QTOF LC-MS	[152]
Amino acids and metabolites in the urea and tricarboxylic acid cycles; biogenic amines; acylcarnitines; lipids, (188, murine tissues).	Absolute IDQ ^TM^ p180 Kit (Biocrates)	LC-MS/MS and FIA-MS/MS, UPLC MS/MS	[153,154]
Essential and non-essential amino acids, phospholipids (32, human breast cancer).	HR MAS	NMR	[155,156]
Identifying, in one session, different classes of compounds from seeds (amygdalin), flowers (rutin), fruits (isovitexin) leaves (shikimic acid) and stems (epicatechin) from Crataegus rhipidophylla Gand (58).	Ratio method	NMR	[157]

**Legend.** *5*-AIQC: 5-aminoisoquinolyl-N-hydroxysuccinimidyl carbamate; BSTFA: N, O-bis-(trimethylsilyl)trifluoroacetamide; GC: gas chromatography; LC: liquid chromatography; MCF: methyl chloroformate derivatization; MS: mass spectrometry; UPLC: ultra performance liquid chromatography; PRM: parallel reaction monitoring; HR MAS—high-resolution magic angle spinning; FIA—flow injection analysis; NMR—nuclear magnetic resonance; QTOF—quadrupole time-of-flight.

## Data Availability

Not applicable.
